# Non-Surgical treatment Versus Surgery for Iatrogenic Femoral Artery Pseudoaneurysms: Systematic Review and Meta-Analysis

**DOI:** 10.3389/fsurg.2022.905701

**Published:** 2022-06-08

**Authors:** Haoliang Wu, Liwei Zhang, Cong Zhang, Boao Xie, Chunyang Lou, Yuanfeng Liu, Hualong Bai

**Affiliations:** ^1^ Department of Vascular and Endovascular Surgery, First Affiliated Hospital of Zhengzhou University, China; ^2^ Key Vascular Physiology and Applied Research Laboratory of Zhengzhou City, China

**Keywords:** surgery, ultrasound guided thrombin injection, ultrasound guided compression, iatrogenic femoral artery pseudoaneurysm, UGTI

## Abstract

**Objectives:**

This study compared results of non-surgical treatment (compression and ultrasound guided thrombin injection (UGTI)) and surgery to treat iatrogenic femoral artery pseudoaneurysms.

**Methods:**

PubMed and Embase databases were searched up to October 2021. Primary outcome measure was success rate, and other outcomes examined were complication rate, reintervention rate. Two authors independently reviewed and extracted data. Data were presented as the odds ratios (ORs) with 95% confidence intervals (CIs). The Grading of Recommendations Assessment, Development and Evaluation (GRADE) approach was used to appraise the quality of the body of evidence.

**Results:**

Eight studies were included. A total of 623 patients with pseudoaneurysm undergoing treatment were included, of which 163 subjects underwent surgery, 397 subjects underwent compression, and 63 subjects underwent UGTI. The success rate was significantly lower in the non-surgery group (OR 0.24, 95% CI, 0.08–0.69, *I*^2 ^= 0%). The complication rate was significantly lower in the non-surgery group (OR 0.10, 95% CI, 0.03 –0.29, *I*^2 ^= 0%). Patients in the non-surgery group tended to have a lower, but statistically insignificant, reintervention rate (OR 0.11, 95% CI, 0.01–1.06, *I*^2 ^= 35%). Further, the GRADE assessment showed that these results (success rate, complication rate, and reintervention rate) were of very low quality.

**Conclusions:**

Available evidence shows that it is reasonable to regard non-surgical treatment as the primary treatment for iatrogenic femoral artery pseudoaneurysms, and surgery as a remedy after failure of non-surgical treatment in some cases.

## Introduction

Currently, in the diagnostic and therapeutic procedures of coronary and peripheral circulation, percutaneous arterial puncture is very common. With development of minimally invasive treatment technology, endovascular techniques have become the first line treatment option for many diseases (1, 2). The femoral artery is the most common site of access, so iatrogenic pseudoaneurysm is common in the femoral artery (3, 4). Pseudoaneurysm is formed when the puncture site is not fully sealed and leading to arterial rupture into the surrounding tissues (5, 6). It is associated with the characteristic manifestations of pulsatile masses, palpable tremors, and audible to-and-fro murmur (4). And it is one of the most common postoperative complications (7, 8), which is associated with the length of operation, the diameter of the catheter, antiplatelet agents, anticoagulation, older age, obesity, ineffective periprocedural compression, hypertension, peripheral arterial disease, hemodialysis and the puncture site (4, 8).

Surgical repair has been the traditional therapy (9). Non-surgical techniques were commonly used to treat pseudoaneurysm including compression and ultrasound-guided thrombin injections (UGTI). The main purpose of treatment is to prevent rupture and relieve the compression symptoms of veins, nerves, and skin (10, 11).

For asymptomatic patients with small pseudoaneurysms (<2 cm), be alert and wait for spontaneous closure is appropriate (8). For large (≥2 cm) and/ or symptomatic pseudoaneurysms, the risks of rupture and bleeding are higher if left untreated (10, 12). And it may cause other complications such as deep vein thrombosis(DVT), neuropathy, and skin necrosis (8, 10, 12). Although endovascular therapy (covered stents and coil embolization) and ultrasound-guided glue injection are currently used in the treatment of pseudoaneurysm, their efficacy and safety have only been demonstrated in a small population, and large studies are needed to confirm this (13, 14). Surgical treatment was the main treatment for iatrogenic femoral artery pseudoaneurysms. In the decade of the 1990s, the transition from surgical treatment to ultrasound-guided therapy initially using noninvasive compression was gradually completed. Recently, UGTI has been widely used as a new minimally invasive technique (15).

This systematic review and meta-analysis aimed to analyze the clinical outcomes of different techniques (surgery and non-surgery) for the treatment of iatrogenic femoral artery pseudoaneurysm and report outcomes including success rate, incidence of complication, and reintervention rate.

## Method

This systematic review and meta-analysis was conducted based on the Preferred Reporting Items for Systematic reviews and Meta-Analysis (PRISMA) Statement (16, 17) and designed according to the latest methodological guidance (18).

### Eligibility Criteria

The current analysis included original research studies that reported outcomes of non-surgery (compression or UGTI) and surgery for treatment of iatrogenic femoral artery pseudoaneurysms. Studies considered for inclusion met the following criteria: Each study had to report on the outcomes of the treatment modalities; femoral artery pseudoaneurysms were objectively diagnosed. Change from one to the other technique, in case of failure, within each study was allowed and was not used as an exclusion criterion. Studies on traumatic pseudoaneurysms and reports examining only one technique were excluded, as were studies whose data was incomplete and case reports. Studies reporting other techniques of femoral artery pseudoaneurysms treatment like para-aneurysmal saline injection, endovascular coils, and ultrasound-guided glue injection, etc., also were discarded.

### Search Strategy

Database search was updated last on October, 2021 in the PubMed and Embase. No restriction on language or publication period was required. Search terms included “femoral artery pseudoaneurysm”, “iatrogenic femoral artery pseudoaneurysm”, “ultrasound guided thrombin injection”, “UGTI”, “ultrasound guided compression”, “UGC”, “compression” and “surgery”. Moreover, we enriched our search by manually reviewing the reference lists of all retrieved articles for additional published or unpublished trials.

### Study Selection

Three review authors screened the titles and abstracts of each search result for potentially relevant studies independently. Then the selected articles were read in full to review studies eligibility and quality. Disagreements were resolved by consensus if necessary. All studies were required to directly report the results of each procedure.

### Data Extraction and Management

Two review authors independently extracted data from each included study using standard forms. Data for all relevant outcomes were extracted from retrieved studies. We collected the following data: number, sex and age of enrolled patients, types of studies, number of patients with pseudoaneurysm in each treatment, and type of intervention. The primary endpoint of the analysis was success rate of treatment modalities. Successful treatment was defined as thrombosis of the flow lumen. Surgical failure was defined as postoperative death of the patient. Secondary endpoints included complication rate, reintervention rate.

### Assessment of Methodological Quality

The quality of each study was assessed based on well-established criteria (Newcastle-Ottawa scale) for non-randomised studies regarding selection (0–4 points), comparability (0–2 points) and identification of the exposure of study participants (0–3 points) independently. We evaluated quality base on it with discrepancies resolved by a third author. The Grading of Recommendations Assessment, Development and Evaluation (GRADE) approach was used to evaluate the quality of evidence (19). One of four grades of very low, low, moderate, and high represented the results of the GRADE approach.

### Statistical Analysis and Data Synthesis

All analyses were conducted using Review Manager (RevMan, version 5.3. Copenhagen: The Nordic Cochrane Centre, The Cochrane Collaboration, 2012). The Mantele-Haenszel method was used to combine summarized data from each report. According to the data collected, we generated odds ratios (ORs) and 95% confidence intervals (CIs) for success rate, complication rate and reintervention rate. The software produced forest plots, and provided inconsistency (*I*^2^) statistics to evaluate the heterogeneity of the included studies. For inconsistency variable a value of 0% indicates no heterogeneity, whereas increasing values suggest increasing heterogeneity. To exclude possible bias because of the quality of the included studies, a random effects model to make comparisons was preferred over a fixed effects model. Publication bias was assessed by generating funnel plots.

## Result

### Literature Search

**Figure 1:**
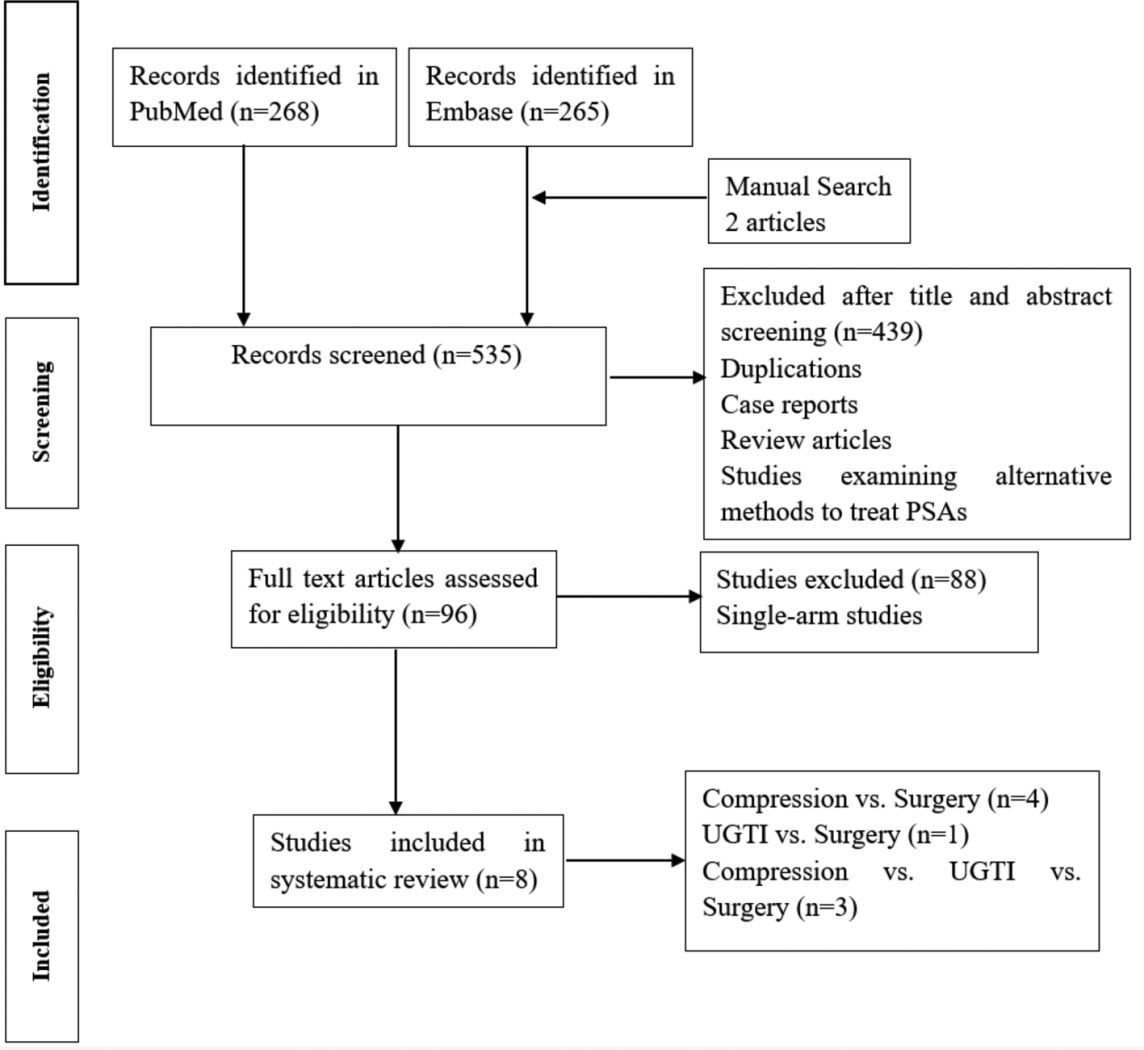
Literature search flow-chart.

The database search returned 533 results. And there were two additional articles that were identified through the manual search of references of primary articles. After screening the title and abstract, 439 studies were excluded. The excluded reports included case reports, review articles, and studies examining alternative methods to treat pseudoaneurysms (para-aneurysmal saline injection, endovascular coils, and ultrasound-guided glue injection), which were not the focus of this analysis. Then, by reading the full texts, 88 studies were excluded because they reported on single-arm treatment (compression or UGTI). Eight studies that finally met the inclusion criteria were selected. The flow chart of the selection procedure is shown in Figure 1.

### Study Characteristics and Quality

Eight retrospective studies were identified and included in this report (20–27). Tables 1, 2 showed the baseline characteristics of the patients included in our analysis. The number of patients ranged from 17 to 227. These studies were published between 1997 and 2020. A total of 623 patients with pseudoaneurysm undergoing treatment were included, of which 163 subjects underwent surgery, 397 subjects underwent compression (344 ultrasound guided compression, 39 compression bandage, 14 manual compression), and 63 subjects underwent UGTI. Moreover, 35 subjects in the former group switched to surgery after unsuccessful compression, 1 subject in the former group switched to surgery after unsuccessful UGTI, 1 subject in the former group switched to surgery after unsuccessful observation, for a total of 163 + 35 + 1 + 1 = 200 subjects undergoing surgery. In addition, one subject in the former group switched to UGTI after unsuccessful compression, for a total of 63 + 1 = 64 subjects undergoing UGTI. It can be seen that most pseudoaneurysms occurred after femoral cannulation for cardiac procedures.

**Table 1:** Overall characteristics of the studies.

Study	Study design	Total patients	Age	Male (%)	Surgery	Non-surgery	Type of intervention	
							Cardiac	Periphera
Huseyin, 2013	Retrosp	55	60.7 ± 6.3	29.1	42	13	55	0
Saydam, 2020	Retrosp	55	NA	NA	31	24	46	6
Savolainen,2012	Retrosp	122	71	NA	33	89	78	44
Heis, 2008	Retrosp	25	NA	NA	8	20	0	25
Stone, 2016	Retrosp	36	66 ± 11	46	18	19	37	0
Demirbas, 2005	Retrosp	17	NA	NA	7	17	NA	NA
Zahn, 1997	Retrosp	86	63 ± 69.7	48	23	86	86	0
Ohlow, 2009	Retrosp	227	NA	NA	38	193	227	0

**Table 2:** Supplementary characteristics of the studies.

Study	Artery	Diagnosis	Antiplatelet or Anticoagulant	Neck diameter (mm)	Neck length (mm)	Sheath size
Huseyin, 2013	SFA 29 CFA 9 DFA 4 Unkonwn 13	Doppler ultrasonography and MRA	NA	NA	NA	NA
Saydam, 2020	NA	CDU	31	2.55:3.04	8.58:9.33	NA
Savolainen, 2012	NA	NA	NA	NA	NA	NA
Heis, 2008	SFA 5 CFA 21 Unkonwn 3	CDU	23	NA	NA	6–9.5F
Stone, 2016	NA	Doppler ultrasonography	65	NA	NA	4–7F
Demirbas, 2005	NA	CDU	NA	NA	NA	NA
Zahn, 1997	NA	Doppler ultrasonography or CDU	86	NA	NA	5–7F
Ohlow, 2009	SFA 41 CFA 104 DFA 82	Doppler ultrasonography	NA	NA	NA	NA

*CDU, color Doppler ultrasonography; CFA, common femoral artery; DFA, deep femoral artery; MRA, magnetic resonance angiography; SFA, superficial femoral artery.*

The median Newcastle-Ottawa scale score was 7 stars (range 5–9). In general, low quality articles had increased risk in their data analysis and confounding (Table 3).

**Table 3:** Quality appraisal checklist for the included studies.

Study	Selection				Comparability	Outcome		
	Representativeness of the Exposed Cohort	Selection of the Non-Exposed Cohort	Ascertainment of Exposure	Demonstration That Outcome of Interest Was Not Present at Start of Study	Comparability of Cohorts on the Basis of the Design or Analysis	Assessment of Outcome	Was Follow-Up Long Enough for Outcomes to Occur	Adequacy of Follow Up of Cohorts
Huseyin, 2013	*	*	*			*	*	*
Saydam, 2020	*	*	*	*	**	*	*	*
Savolainen,2011	*	*	*			*	*	*
Heis, 2008	*	*	*	*		*	*	*
Stone, 2016	*	*	*			*	*	*
Demirbas, 2005	*	*				*	*	*
Zahn, 1997*	*	*				*	*	*
Ohlow, 2009	*	*		*		*	*	*

### Data Synthesis

**Figure 2:**
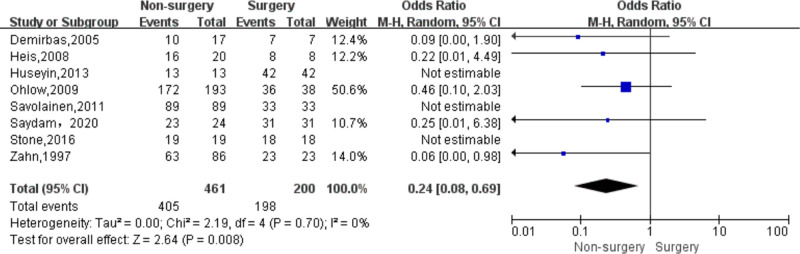
Forest plot showing the pooled odds ratio for pseudoaneurysm treatment success rate after surgery and non-surgical treatment.

**Figure 3:**
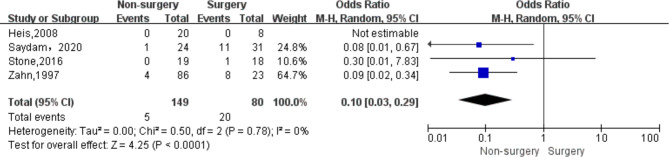
Forest plot showing the pooled odds ratio for pseudoaneurysm treatment complication rate after surgery and non-surgical treatment. CI, confidence interval; IV, inverse variance.

**Figure 4:**
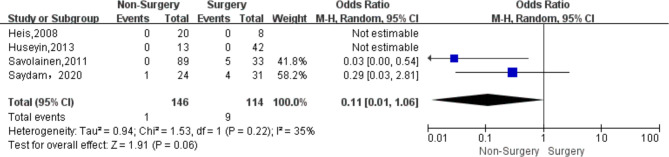
Forest plot showing the pooled odds ratio for pseudoaneurysm treatment reintervention rate after surgery and non-surgical treatment. CI, confidence interval; IV, inverse variance.

**Figure 5:**
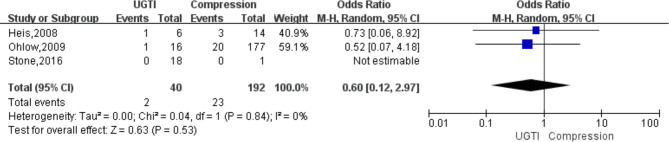
Forest plot showing the pooled odds ratio for failure rate of subgroup analysis about compression and UGTI group.

Data regarding efficacy of strategies to treat iatrogenic femoral artery pseudoaneurysms could be extracted from all 8 included studies. In total 200 patients underwent surgery and 461 non-surgical treatments (Table 4). The success rate was significantly lower in the non-surgery group (OR 0.24, 95% CI, 0.08–0.69, *I*^2 ^= 0%) (Figure 2). With respect to safety, there were low complication rates for both groups. Data was available for 200 patients undergoing surgery, and corresponding complication rates were 10.0% (20/200). There were 461 patients in the non-surgical group, with a total complication rate of 1.1% (5/461), of which the complication rate in the compression group was 1.0% (4/397) and the complication rate in the UGTI group was 1.6% (1/64). Among patients in the surgery group, there were six cases of surgical site infection, six cases require wound drainage (cause unknown), three cases of major hemorrhage, two cases of maceratin, one case of DVT, one case of sepsis, and one case of arteriovenous fistula. Among patients in the compression group there was one case of DVT, one case of arterial occlusion, one case of local infection, and one case of major hemorrhage. Among patients in the UGTI group there was one case of thrombin emboli. The complication rate was significantly lower in the non-surgery group (OR 0.10, 95% CI, 0.03–0.29, *I*^2 ^= 0%) (Figure 3). Patients in the non-surgery group tended to have a lower, but statistically insignificant, reintervention rate (OR 0.11, 95% CI, 0.01–1.06, *I*^2 ^= 35%) (Figure 4). Four of the patients who underwent reintervention after surgery had hematomas and five had wound infections. Subgroup analysis result showed that the failure rate of compression and UGTI group was not significantly different (OR 0.60, 95% CI, 0.12–2.97, *I*^2 ^= 0%) (Figure 5).

**Table 4:** Summary of outcomes.

Intervention	Total cases	Success rate (%)	Complications (%)	Reintervention (%)
Surgery	200	198 (99.0)	20 (10.0)	9 (4.5)
Compression	397	344 (86.6)	4 (1.0)	0 (0)
UGTI	64	61 (95.3)	1 (1.6)	1 (1.6)
Total	661	603 (91.2)	25 (3.8)	10 (1.5)

*UGTI, ultrasound guided thrombin injection.*

### GRADE of Evidence

Based on the results of the GRADE approach, the quality of evidence of outcome measures (success rate, complication rate, and reintervention rate) were rated as very low (Table 5). The large risk of bias was caused by lack of allocation concealment and inadequate blinding. The reason for downgrading for imprecision was the small sample sizes of included studies.

**Table 5:** GRADE evaluation of evidence quality.

								Number of patients		Effect		
Outcome	No. of studies	Study design	Risk of bias	Inconsistency	Indirectness	Imprecision	Other considerations	Non-surgery	Surgery	Relative (95% CI)	Certainty	Importance
Success rate	8	Observational study	Serious^a^	Not serious	Not serious	Not serious	None	461	200	OR0.24 (0.08 to 0.69)	⊕◯◯◯ (VERY LOW)	Critical
Complication rate	4	Observational study	Serious^a^	Not serious	Not serious	Serious^b^	None	149	80	OR0.1 (0.03 to 0.29)	⊕◯◯◯ (VERY LOW)	Critical
Reintervention rate	4	Observational study	Serious^a^	Not serious	Not serious	Serious^b^	None	146	114	OR0.11 (0.01 to 1.06)	⊕◯◯◯ (VERY LOW)	Critical

*CI, confidence interval; OR, odds ratios.*

^a^ *We downgraded for risk of bias, because of lack of allocation concealment and inadequate blinding.*

^b^ *We downgraded for imprecision, because of small sample sizes of included studies.*

### Sensitivity Analysis

Sensitivity analysis was performed to explore the effect of individual studies. The result of success rate produced marked change (OR 0.30, 95% CI, 0.10–0.95, *I*^2 ^= 0%) by excluding the study by Zahn. When analyzing complication rate, sensitivity analysis, results showed no significant change after exclusion of each study.

## Discussion

In this study, we have found significantly higher in the success rate in surgical treatment for iatrogenic femoral artery pseudoaneurysm, but complication rate was significantly higher in the surgery group.

Until the 1990s, surgery was the gold standard treatment for treating iatrogenic femoral artery pseudoaneurysms. Then, UGC (ultrasound guided compression) and UGTI were used to treat pseudoaneurysms (28, 29). Gradually, endovascular treatment (covered stents and coil embolization), para-aneurysmal saline injection, and ultrasound-guided glue injection appeared (13, 30–32). However, each procedure has advantages and disadvantages. In our study, we compared non-surgical and surgical treatments. In the non-surgical treatment group, we only included patients who received compression and UGTI, because no studies comparing other non-surgical treatments with surgical treatment were found.

In our study, the success rate of UGTI was 95.3% and the success rate of compression was 86.6%. And subgroup analysis result showed no significant difference in failure rate. In a meta-analysis comparing UGC and UGTI in 992 patients, it suggested the success rate of UGTI was 97.4% and the success rate of UGC was 69.3% (33). The result about UGTI is similar to our finding. However, there is a major difference in the results of compression. There may be several reasons. In our study, the compression group included patients undergoing manual compression, compression bandage and UGC, while only patients who received UGC were included in the above meta-analysis. The use of anticoagulant and antiplatelet drugs, age, features of pseudoaneurysm and body mass index (BMI) of the enrolled patients were different, and these factors may have a certain impact on the success rate (4, 8). Then, the relatively higher success rate of our study may because of the relatively few patients. Finally, we defined therapeutic success as the final thrombosis of the pseudoaneurysm regardless of whether the compression or UGTI succeed at first time. In some studies, patients had to undergo two or even three attempts of compression before they succeeded (21).

The result we got was that the success rate of non-surgery was significantly lower than that of surgical treatment. It should be noted that a total of 37 patients received surgical treatment after the failure of conservative treatment, including one case of UGTI, one case of observation and 35 cases of compression. There was one surgical failure in these patients. In total, two patients in the surgery group were judged failures due to death, one case was in hemorrhagic shock due to acute rupture of a pseudoaneurysm, and one case was due to multiorgan dysfunction syndrome. The sensitivity analysis about success rate showed marked change by excluding the study by Zahn, which might be related to the fact that it was an earlier study and only it included patients treated by different compression methods (compression bandage and UGC) (27).

For the comparison of two non-surgical treatment methods, a meta-analysis involving 996 patients pointed out that UGTI is better than UGC (33). This study pointed out that UGTI resulted in a higher success rate in the treatment of pseudoaneurysms, and might be associated with lower complication rate and treatment cost.

There were many complications of surgery and compression, while in the UGTI group, according to our results, only one complication was thrombin emboli (25). At present, one study has shown that thrombin emboli risk may be negatively correlated with sac area and neck length, and negatively correlated with neck width (34). However, another study suggested that pseudoaneurysm neck dimensions was not associated with treatment efficacy (35). Skin infection is another potential complication of UGTI identified by Weinmann et al. (36) The fatal complication of UGTI is an anaphylactic reaction to thrombin, which is related to the use of bovine thrombin and repeated exposure to thrombin (37).

Currently, UGTI is often the first-line treatment for iatrogenic post-catheterization pseudoaneurysms. But there are also treatments that use various glues instead of thrombin, such as N-butyl cyanoacrylate (NBCA) (38), N-butyl cyanoacrylate-methacryloxy sulfolane (NBCA-MS) (39), fibrin glue (40), and tissue glue (TG) (41). The use of these glues has been proven to be safe and effective, and they have the potential to become an alternative to thrombin (14).

This meta-analysis also has some limitations. Firstly, the quality of the studies we included was not high, which contributed to the very low quality of our evidence. Second, the final results were combined using a random-effects model, and the weight of each article was proportional to the number of cases reported, so the source of variation should be considered within the study. There may also be some deviations in some cases, because the circumstances of each study are different, the basic characteristics of the patients and the characteristics of pseudoaneurysms are different. In addition, the current study was not registered, so there might be a small bias, but we still strictly follow the steps of systematic review. Last, in the current protocol, crossover from compression or UGTI to surgery is allowed, which may have some impact on the outcome.

## Conclusion

The success rate of surgery of iatrogenic femoral artery pseudoaneurysm is higher than that of non-surgical treatment, but the complication risk is higher, and the reintervention rate also tends to be higher. Therefore, it is reasonable to regard non-surgical treatment for pseudoaneurysm as main treatment if the situation is not critical, and surgical treatment as a remedy after failure. Moreover, the quality of the success rate, complication rate, and reintervention rate assessments was very low. Further research is required because of the low overall quality of the included studies.

## Data Availability

The original contributions presented in the study are included in the article/Supplementary Material, further inquiries can be directed to the corresponding author/s.
